# Analyses of Human Genetic Data to Identify Clinically Relevant Domains of Neuroligins

**DOI:** 10.3390/genes15121601

**Published:** 2024-12-14

**Authors:** Alexander W. Lehr, Kathryn F. McDaniel, Katherine W. Roche

**Affiliations:** 1 Receptor Biology Section, National Institute of Neurological Disorders and Stroke, National Institutes of Health, Bethesda, MD 20892, USA; alexander.lehr@nih.gov (A.W.L.); kate.mcdaniel@nih.gov (K.F.M.); 2 Department of Neuroscience, Brown University, Providence, RI 02906, USA

**Keywords:** neuroligin, autism spectrum disorder, neurodevelopmental disorder, human genetic variation, missense variation, haploinsufficiency

## Abstract

**Background/Objectives:** Neuroligins (NLGNs) are postsynaptic adhesion molecules critical for neuronal development that are highly associated with autism spectrum disorder (ASD). Here, we provide an overview of the literature on *NLGN* rare variants. In addition, we introduce a new approach to analyze human variation within *NLGN* genes to identify sensitive regions that have an increased frequency of ASD-associated variants to better understand NLGN function. **Methods**: To identify critical protein subdomains within the *NLGN* gene family, we developed an algorithm that assesses tolerance to missense mutations in human genetic variation by comparing clinical variants from ClinVar to reference variants from gnomAD. This approach provides tolerance values to subdomains within the protein. **Results**: Our algorithm identified several critical regions that were conserved across multiple NLGN isoforms. Importantly, this approach also identified a previously reported cluster of pathogenic variants in *NLGN4X* (also conserved in *NLGN1* and *NLGN3*) as well as a region around the highly characterized NLGN3 R451C ASD-associated mutation. Additionally, we highlighted other, as of yet, uncharacterized regions enriched with mutations. **Conclusions**: The systematic analysis of NLGN ASD-associated variants compared to variants identified in the unaffected population (gnomAD) reveals conserved domains in NLGN isoforms that are tolerant to variation or are enriched in clinically relevant variants. Examination of databases also allows for predictions of the presumed tolerance to loss of an allele. The use of the algorithm we developed effectively allowed the evaluation of subdomains of NLGNs and can be used to examine other ASD-associated genes.

## 1. Introduction

Neurodevelopmental disorders (NDDs) are a group of conditions characterized by impairments that arise during brain development, such as autism spectrum disorder (ASD), intellectual disability (ID), developmental delay (DD), and epilepsy. Often these NDDs have a high rate of comorbidity, suggesting common underlying etiological factors [1,2,3]. Next-generation sequencing (NGS) techniques, such as whole genome/exome sequencing (WGS/WES), have greatly increased the number of identified genetic variants, both benign and disease-associated. With the availability of larger human genetic datasets, researchers have been able to home in on specific commonly mutated gene families that are causal for disease. Increasingly, there have been large efforts in not only sequencing clinical patients, but also sequencing large cohorts of undiagnosed individuals, with presumably tolerant sets of mutations. This has revealed that NDDs have convergent and comorbid phenotypes, suggesting a convergence in disrupted molecular networks/pathways.

For scale, there are around 3 billion base pairs in the human genome, and an estimated rate of 1 reported variant per every 1000 base pairs (i.e., for every 1000 base pairs in a sequenced subject, 1 of those base pairs will differ from the established reference human genome), totaling around 2 million variants within one sequenced individual [4,5,6]. On average, there are ~20,000 variants found within exomic coding regions, with ~500 of those being rare missense variants. Ultimately, any given genome likely includes 1–2 de novo coding mutations [4,5]. The large number of identified variants leads to vastly expanding databases and an ever-growing challenge to completely understand how genetic risk can lead to disease.

One commonly disrupted protein network in NDDs includes genes encoding synaptic proteins such as those mediating synaptic contact/adhesion, synaptic vesicle release, post-synaptic scaffolding, and receptor localization and function [2,7]. ASD-associated missense mutations disrupt receptor/channel function, binding interfaces, or post-translational modification sites such as phosphorylation or glycosylation, abolishing the temporal and spatial control these domains exert over the protein, and their broader signaling pathways [8,9,10,11,12].

Neuroligins (*NLGN*s) were some of the earliest identified ASD-associated risk genes, along with their presynaptic binding partners neurexins (NRXNs) [13,14]. NLGNs are single-pass transmembrane proteins consisting of a large acetylcholinesterase-like extracellular domain and a short intracellular tail [15]. They bind to NRXNs, a family of presynaptic proteins, in a calcium-dependent manner to promote synapse specification, formation, and maintenance [16,17,18]. All NLGNs contain the same major conserved domains (NRXN binding domain, gephyrin binding domain, PDZ-domain binding motif) and have 65% or higher sequence homology between all five human *NLGN* genes [19]. *NLGN1* and *NLGN2* are autosomal, located on the 3rd and 17th chromosomes, respectively. *NLGN3* and *4X* are located on the X chromosome, *NLGN4Y* on the Y chromosome, with *NLGN4X* and *4Y* arising recently in mammalian evolution, falling out of the pseudo-autosomal region of the sex chromosomes [19,20].

Many of the ASD-associated mutations on *NLGNs* affect their synaptogenic properties (often experimentally observed as fewer spines and/or weaker post-synaptic potentiation), either through early truncating variants, resulting in the potential loss of function (pLoF), or missense mutations that disrupt functional protein domains. These missense mutations can disrupt NLGN dimerization, trafficking, or binding to NRXNs and in some cases, alter post-translational modifications of NLGNs [13,21,22,23,24,25,26,27,28,29,30,31]. For example, an ASD-associated variant in NLGN4X’s intracellular tail blocks phosphorylation of a nearby residue, resulting in robust functional consequences [21]. In contrast, in the extracellular domain of NLGN4X, a cluster of ASD-associated mutations near a crucial glycosylation site disrupts normal surface trafficking of the protein and results in decreased excitatory, but not inhibitory synapse formation [32]. As more clinically relevant point mutations are identified near distinct structural and functional domains, researchers will be able to predict the importance of these features just through examining the human genetic data allowing for more targeted hypothesis testing. In this manuscript, we provide a systematic examination of sensitive domains within NLGNs by comparing disease-associated human genetic databases to human genetic databases of unaffected individuals. We specifically discuss several new clusters of ASD-associated variants in the *NLGN* genes based on database comparisons.

## 2. NLGNs in NDDs

### 2.1. NLGN1 in NDDs

Both nonsense and missense variants in *NLGN1* have been identified in patients with ASD (Table 1). NLGN1 p.L25*, a nonsense truncating variant, was identified in monozygotic twins with ASD, who were homozygous for the variant. This rare genotype resulted from the children inheriting one copy of the variant from each parent, who are first cousins. The parents are unaffected, suggesting a dose-dependent effect of this variant, which likely results in a complete loss of NLGN1 protein due to nonsense-mediated decay (NMD) of message or destabilization of the truncated protein following translation [33].

NLGN1 p.P89L is a well-characterized missense variant that was identified in two brothers, both with ASD, who inherited it from an unaffected mother presumed to be a carrier [34]. NLGN1 p.P89L displays reduced neuronal surface expression and decreased spine density. Similarly, NLGN1 p.P89L exhibits reduced synaptogenic activity in a co-culture assay when expressed in African green monkey kidney cells (COS7) and incubated with neurons as compared to NLGN1 wild-type (WT) [34,35]. A knock-in (KI) mouse model of NLGN1 p.P89L demonstrated a 30% decrease in NLGN1 protein expression in the heterozygous mice, and a 50% decrease in NLGN1 protein expression in the homozygous mice. Heterozygous, but not homozygous, mice showed aberrant social behavior in the three-chamber test and caged social interaction test, whereas both heterozygous and homozygous mice showed spatial memory deficits [34]. Differences between heterozygous and homozygous KI behavior highlights the importance of studying accurate disease models, as relatively minor differences in gene expression or function can lead to functional consequences.

Multiple additional *NLGN1* missense variants have been reported in the literature. NLGN1 p.D140Y, p.T90R, and p.I158K were identified in patients with speech disorders, DD, ASD, ADHD, and/or ID [36,37]. The p.T90 residue was previously identified as a missense variant (p.T90I) in a different cohort of patients [34] and showed no difference in expression levels, ER-retention, or spine density as compared to WT NLGN1. Three NLGN1 missense variants (p.L269P, p.G297E, and p.H795Y) display impaired surface expression and reduced spine number in neurons as compared to WT NLGN1 [34].

Outside of NDDs, there is a well-characterized *NLGN1* variant that was found in a patient with Alzheimer’s disease. NLGN1 p.T271fs is a frameshift variant that results in a premature stop codon [38]. Studies on NLGN1 p.T271fs homozygous mice reveal reduced truncated transcript expression and almost no protein expression, whereas heterozygous mice have a 50% reduction in NLGN1 protein expression [39]. Behaviorally, heterozygous mice have recognition memory deficits and age-dependent deficits in long-term potentiation compared to homozygous mice, which exhibit neither of these phenotypes [39]. These results emphasize the importance of the study of disease-specific variants in animal models to most accurately model cellular and behavioral effects and to help advance a search for new therapeutics.

**Table 1:** Summary of reported NLGN1 variants.

Gene	Variant	Patient Clinical Presentation	Predicted Loss of Allele	Functional Effects in Culture	Mouse Model Phenotypes	Citation
NLGN1	L25*	ID/ASD	Yes		-	[33]
	P89L	ASD	No	Decreased spine number; ER-retained; decreased synapse number in co-culture	Decreased protein expression; spatial learning and social deficits	[34,35]
	T90I	ASD	No	Unaffected surface expression and spine number	-	[34]
	T90R	Speech disorder	No	-	-	[37]
	D140Y	ASD/ADHD	No	-	-	[36]
	I158K	ID/DD	No	-	-	[37]
	L269P	ASD	No	Decreased spine number; ER-retained	-	[34]
	T271fs	AD	Yes	-	Decreased protein expression; age-dependent memory deficits	[38,39]
	G297E ^^^	ASD	No	Decreased spine number; ER-retained	-	[34]
	H795Y ^^^	ASD	No	Decreased surface expression; decreased spine number	-	[34]

^^^ NLGN1: G297E is G288E in mice; H795Y is H786Y in mice.

### 2.2. NLGN2 in NDDs

*NLGN2* currently has 167 reported variants in ClinVar, with 72 missense variants, and 8 reported early truncation variants. Additionally, a de novo nonsense variant, NLGN2 p.Y147*, was identified in a patient with ASD and ID [40], but is not reported in ClinVar (Table 2). The patient was also diagnosed with hyperphagia, obesity, and obsessive and self-injurious behaviors often related to food. The functional consequences of this variant were not evaluated in vitro, although the variant was predicted to result in haploinsufficiency due to being an early truncation before the last exon and thus presumed to undergo NMD [40].

In addition to NLGN2 p.Y147*, duplication events including *NLGN2* have been reported in patients with ID [41,42], and there are nine copy number variations involving *NLGN2* reported in ClinVar. While *NLGN2* is a gene of interest due to the known ASD-association of other *NLGNs*, the effect of the *NLGN2* duplication is unclear, as *NLGN2* was one of many genes affected by each duplication [41,42].

The most well-characterized *NLGN2* variant was identified in a genetic screen of a cohort of schizophrenia patients in Taiwan, which identified four *NLGN2* missense variants (p.R215H, p.V510M, p.R621H, p.A637T) that were not present in controls [23]. From this screen, NLGN2 p.R215H was the only one of the four variants that showed increased ER-retention and decreased GABAergic synaptogenesis and function [23]. A KI mouse model of the NLGN2 p.R215H variant exhibited an approximately 40% or 90% reduction in NLGN2 protein in heterozygous and homozygous KI mice, respectively [24]. Homozygous NLGN2 p.R215H mice displayed reduced levels of inhibitory synaptic proteins, a decrease in mIPSC amplitude and frequency, and an altered E/I ratio [24,25]. Behaviorally, NLGN2 p.R215H mice displayed increased anxiety-like behavior and a reduced pre-pulse inhibition [24,25]. Importantly, the NLGN2 p.R215H mouse displays different phenotypes than the *NLGN2* KO mouse [24,43]. These differences emphasize the need for specific patient variant mouse models, especially in heterogeneous conditions such as psychiatric disorders and NDDs.

### 2.3. NLGN3 in NDDs

NLGN3 and NLGN4X were the first NLGNs to be identified with variants in ASD patients (Table 3) [13]. Two affected brothers in a Swedish family were found to have a NLGN3 p.R451C variant, with an unaffected mother carrier [13]. Early biochemical characterization of NLGN3 p.R451C revealed impaired surface expression, increased ER-retention, and reduced co-localization with synapsin as compared to NLGN3 WT when expressed in either heterologous cell cultures or hippocampal neurons [22,26,44]. In co-cultures of HEK293T cells expressing NLGN3 WT or p.R451C with either rat or hiPSC-derived neurons, NLGN3 p.R451C significantly reduced pre-synaptic formation in neurons, as measured by synapsin fluorescence [45]. However, NLGN3 p.R451C can still form homodimers and heterodimers with NLGN1, which occurs in the ER and is required for surface expression, suggesting that the mechanism of NLGN3 p.R451C ER-retention is not disrupted dimerization [46].

As the first NLGN missense variant identified, the NLGN3 p.R451C variant is the most well-characterized in animal models of ASD-associated NLGN variants. NLGN3 R451C KI mice show a 90% reduction in the expression of NLGN3 in hemizygous male mice, as compared to NLGN3 WT males [27]. In studies of NLGN3 R451C KI mice, region-specific synaptic and electrophysiological phenotypes were revealed. For example, in layer 2/3 of the somatosensory cortex, NLGN3 R451C KI synapses are morphologically unaffected but there is an increase in mIPSC frequency and evoked IPSC amplitude, whereas excitatory synaptic measures are unchanged [27]. In contrast to findings in the somatosensory cortex, in the hippocampus of NLGN3 R451C KI animals there is a decrease in synaptic terminal and spine size, along with an increase in excitatory measures, such as fEPSP slope and AMPAR/NMDAR ratio, which is not seen in the WT or KO NLGN3 lines [47]. The NLGN3 R451C KI mouse also displayed an increased NMDAR decay time [47]. Other circuit-specific changes have been investigated in the Calyx of Held synapses, endocannabinoid circuits in the hippocampus, and the somatosensory cortex [48,49,50].

Multiple NLGN3 R451C KI lines have been created or backcrossed onto different genetic backgrounds [27,28,51]. The majority of NLGN3 p.R451C characterization has been done with the original KI line [27]. There is one study comparing excitatory synaptic measures in two different NLGN3 R451C KI lines which revealed no difference between the two lines [47]. In behavioral testing, NLGN3 R451C KI mice display decreased social behavior (e.g., novel object test) and enhanced learning (e.g., Morris water maze and rotarod) [27,47,52]. These behavioral phenotypes appear to be affected by genetic background, with no change in some [51] and a loss of ASD-like behaviors in others [28]. These variations underscore the importance of variation in genetic background and methodology between labs. NDDs, such as ASD, are notoriously heterogenous, and while differences in findings might be difficult to reconcile, they are likely representative of the heterogeneity of phenotypes that are seen in human patients with the same mutation.

Two other NLGN3 missense variants, p.R597W and p.P514S, have been identified in multiple probands in two different families [53,54]. NLGN3 p.R597W and p.P514S both display reduced surface expression and increased ER-retention; furthermore, overexpression of the mutated protein leads to increased levels of ER stress proteins, such as BiP and CHOP [53]. Three additional NLGN3 missense variants, NLGN3 p.V184A, NLGN3 p.G426S, and NLGN3 p.V321A, have been identified in patients with ASD, none of which have been characterized further [55,56,57]. Two NLGN3 truncation variants, NLGN3 p. W122* and p.R55*, were identified in patients with both ASD and Gonadotropin-Releasing Hormone Deficiency (GD) [58]. Thorough characterization of these mutations under the endogenous promoter to determine if it undergoes NMD has yet to be reported.

**Table 3:** Summary of reported NLGN3 variants.

Gene	Variant	Patient Clinical Presentation	Predicted Loss of Allele	Functional Effects in Culture	Mouse Model Phenotypes	Citation
NLGN3	R55*	ASD, GD	Yes	-	-	[58]
	W122*	ASD, GD	Yes	-	-	[58]
	V184A	ASD	No	-	-	[57]
	V321A	ASD/ID, hearing loss, obesity	No	-	-	[56]
	R451C	ASD	No	Increased ER-retention; impaired synaptogenesis	Decreased protein expression; circuit-specific changes in synapse function; background-dependent changes in social and anxiety-like behaviors	[13,22,26,27,28,44,45,47,52,59]
	G426S	ASD	No	-	-	[55]
	P514S	ASD/ID, language impairments	No	Impaired surface trafficking; UPR upregulation	-	[53]
	R597W ^^^	ASD/ID	No	Impaired surface trafficking; UPR upregulation	-	[53,54]

^^^ NLGN3: R597W was identified as R617W in the initial publication.

### 2.4. NLGN4X in NDDs

As NLGN4X and 4Y in humans are not highly conserved with NLGN4-like in rodents, the study of disease-related NLGN4 mutations in model systems is not straightforward. There are several studies of NLGN4-like KO animals, which display deficits including ASD-associated behavioral phenotypes [60,61]. Due to the divergence in sequence between rodent and human, many labs have begun using human-derived iPSCs to model NLGN4-related ASD-associated dysfunction.

Both deletions and duplications from large chromosomal abnormalities and those specific to NLGN4X or NLGN4Y have been identified in patients of both sexes with NDDs (Table 4). The precise disease etiology of broad deletions is difficult to discern, as there are often many genes involved, as well as carrier family members with no or different disease presentation [62,63,64,65,66,67,68,69,70]. However, reports of NDDs in patients with NLGN4X-specific truncations [13,14], functional duplications of NLGN4X [71], and sex chromosome trisomies [72,73,74] indicate that physiological NLGN4X and NLGN4Y expression levels are required for proper neurodevelopment.

Multiple missense variants in NLGN4X have been identified in patients with ASD and other NDDs. These variants include p.G84R, p.R87W, p.P94L, p.G99S, p.R101Q, p.V109L, p.A283T, p.Q162K, p.K378R, p.V403M, p.L593F, p.R704C, and p.T787M [21,29,30,55,75,76,77,78]. Variants were found in both males and females and have varying penetrance among other family members. Broadly, these variants display impaired protein maturity, synaptogenesis, and surface expression [21,30].

NLGN4X p.R87W was identified in two brothers with ASD, but was not found in either parent [29]. Biochemical characterization of NLGN4X p.R87W demonstrates decreased surface expression, decreased synaptic density, and a failure to suppress excitatory synaptogenesis that is seen with NLGN4X WT expression [29]. NLGN4X p.R704C was first identified in a male patient with dizygotic female twin sisters, one with an undescribed developmental disorder not carrying the mutation, with the other unaffected sister carrying the heterozygous allele, leaving the penetrance of this variant inconclusive [75]. NLGN4X p.R704C inhibits PKC-mediated NLGN4X phosphorylation at p.T707, which, under WT conditions, leads to an increase in synaptogenesis and NMDAR- and AMPAR-mediated excitatory activity in rodent hippocampal cultures [31]. Expression of NLGN4X p.R704C in neurons increases synaptic density and leads to an increase in eEPSC amplitude and enhanced binding to GluA1 compared to NLGN4X WT [79].

NLGN4X p.R101Q was identified in a patient with ASD and resides in a sensitive region clustered with other pathogenic ASD-associated variants, all of which have surface trafficking deficits [21]. NLGN4X p.R101Q displays reduced surface expression and is ER-retained. Additionally, NLGN4X p.R101Q has reduced glycosylation at site p.N102 [32]. The “hotspot” of mutations around p.R101Q spans from amino acids 75–125 in NLGN4X, and there is a corresponding deficit of variation found in the unaffected population. All disease-associated variants in this hotspot lead to decreased NLGN4X maturation, and the two variants that underwent electrophysiological analyses (p.P94L and p.G99S) lead to decreased mEPSC frequency and amplitude. When aligned with NLGN4Y, there are three amino acid differences in this region, including p.P93 (p.S93 in NLGN4Y), which was found to be responsible for the trafficking deficit observed for NLGN4Y. When NLGN4X P93S (a swap to the analogous residue in NLGN4Y) is expressed, it displays decreased surface expression in heterologous cells and neurons [21]. Likewise, a NLGN4Y S93P mutation rescues the NLGN4Y trafficking deficit as compared to WT. A concentration of ASD-related variants in this region suggests that it is a vital domain for NLGN4X and that loss of surface expression is not tolerated in neurodevelopment in males. [21]. Additionally, rescuing surface expression of NLGN4Y could provide a therapeutic approach to NLGN4X mutations in males with ASD.

Only two missense variants in NLGN4Y have been reported in patients with ASD or ID. NLGN4Y p.I679V was identified in a male with ASD, inherited from a father with learning disabilities [80], whereas NLGN4Y p.N163K was identified in three unrelated patients in a population of people with ID [81]. Neither of these variants has been characterized further and more research is needed to understand the implications of these variants.

Thus far, studies on rare variants have originated with cohorts of patients being sequenced, then basic scientists studying one variant at a time. To make the study of ASD-associated genes more systematic, we have developed an algorithm to highlight regions differing in disease populations compared to unaffected populations. In this manuscript, we analyze publicly available human genetic data to identify sensitive regions of *NLGNs*, which are presumed crucial to the structure and function of the protein. Broadly, this algorithm scores genetic variation within a gene and compares benign variants, as listed in gnomAD, to disease-associated variation, as listed in ClinVar. Thus, we can identify sensitive regions that are enriched with disease-associated missense variants, but sparse in well-tolerated missense variants. We developed this method in Python, and the annotated Jupyter Notebooks are publicly available on GitHub.

**Table 4:** Summary of reported NLGN4X and NLGN4Y variants.

Gene	Variant	Patient Clinical Presentation	Predicted Loss of Allele	Functional Effects in Culture	Mouse Model Phenotypes	Citation
NLGN4	G84R	ASD	No	Impaired protein maturity and surface expression; impaired synaptogenesis	-	[21,30,55]
	R87W	ASD	No	Impaired protein maturity and surface expression; impaired synaptogenesis	-	[21,29,30]
	P94L		No	Impaired protein maturity and synaptogenesis	-	[21]
	G99S	ASD/DD	No	Impaired protein maturity and surface expression; impaired synaptogenesis	-	[21,30,75]
	R101Q	ASD	No	Impaired protein maturity and synaptogenesis; impaired glycosylation	-	[21,32]
	V109L	ASD	No	Impaired protein maturity and synaptogenesis	-	[21]
	Q162K	ASD	No	Impaired protein maturity and surface expression; impaired synaptogenesis	-	[30,55]
	A283T	ASD	No	Impaired protein maturity and surface expression; impaired synaptogenesis	-	[30,55]
	K378R	ASD	No	Impaired protein maturity and surface expression; impaired synaptogenesis	-	[30,75,77]
	D396*	ASD	Yes	-	-	[13]
	V403M	ASD	No	Impaired protein maturity and surface expression; impaired synaptogenesis	-	[30,75]
	D429*	ID/ASD	Yes	-	-	[14]
	L593F	ID	No	Impaired protein maturity and surface expression; impaired synaptogenesis	-	[30,76]
	R704C	ASD	No	Affects phosphorylation at T707; increase in excitatory activity	-	[31,75,79]
	T787M	ASD	No	-	-	[76,78]
NLGN4Y	N163K	ID	No	-	-	[81]
	I679V	ASD	No	-	-	[80]

## 3. Results

### 3.1. Description of Relevant Databases

#### 3.1.1. GnomAD

Maintained by the Broad Institute, the Genome Aggregation Database (gnomAD) is a repository of genetic variants from unimpacted populations (i.e., populations without NDDs) that differ from the established consensus human genome (as of this publication, Genome Reference Consortium Human Build 38 [GRCh38]) [4]. With its most recent version 4.1.0, gnomAD is composed of over 730,000 exomic and 76,000 genomic sequences assembled from 308 data contributors (including UK BioBank [82], 1000 genomes project [6], T2T [83]). Use of this database provides powerful insights into the degree of variation within the genome that is tolerated without becoming pathogenic.

It is important to note that there are variants found within gnomAD that are also reported in ClinVar. This means that a variant can occur within an unimpacted person, while also having been identified in an affected individual. These co-occurrences could be due to multiple factors, including genetic background, environment, homo/hemi/heterozygosity, and even diagnostic constraints, highlighting the intricacies of the penetrance of pathogenicity. For example, several studies have examined the functional consequences of an ASD-associated variant on the intracellular CTD of NLGN4X (p.R704C), found in a male patient with ASD with a carrier mother and unimpacted sister [15,79]. This variant was shown to impact NLGN4X p.T704 phosphorylation, as well as excitatory synaptic transmission [31,84]. But with the fourth version of gnomAD, there are an additional 21 individuals with this variant. Although most NLGN4X p.R704C variants found in gnomAD are female heterozygotes (possible carriers), there are three male hemizygotes carrying the mutation as their only copy, thus indicating a preference for female carriers, while some hemizygous males also might tolerate carrying the mutation.

#### 3.1.2. ClinVar

ClinVar acts as a repository containing clinically implicated genetic variants and has been invaluable for connecting clinicians and researchers to investigate potential pathogenic variation. With 925 depositors from 64 countries, the contributors include clinicians, as well as institutes and companies that sequence and study patient populations [85]. Currently, there are over 583,000 submissions in ClinVar. Annotations within ClinVar are provided by the submitters, and reviewers can dispute pathogenic interpretations leading to entries labeled as “conflicting” or “multiple clinical significances” reported [86]. Within these databases, final scores are distilled into a set of five terms established by the American College of Medical Genetics and Genomics and the Association for Molecular Pathology (ACMG/AMP): “pathogenic”, “likely pathogenic”, “uncertain significance”, “likely benign”, and “benign” [87]. Although these terms can be helpful, it is important to realize they broadly simplify what should often be a much more nuanced and deliberate decision in concluding pathogenicity.

### 3.2. Tolerance to Haploinsufficiency in NDDs

#### 3.2.1. Background on Nonsense-Mediated mRNA Decay

NMD is an evolutionarily conserved translation-coupled quality-control mechanism leading to the degradation of mRNA transcripts that contain a premature stop codon. This was first observed in prokaryotic cells with unexpectedly low transcript levels from genes with premature stop codons. In eukaryotes, this phenomenon has been attributed to a set of trans-acting proteins encoded by the UPF1, UPF2, and UPF3 genes, which are evolutionarily conserved. These proteins are believed to form a trimeric complex, called the UPF complex, that directly binds to early-terminating ribosomes, directing degradation of the transcript [88]. Several mechanistic models for how a stop codon is determined to be premature have been posited, including the explanation that the UPF complex detects a premature stop codon via its proximity to the poly(A) binding protein bound to the 3′ UTR [88].

The prevalence of NMD means that early truncation mutations (a stop codon occurring before the last exon) likely result in reduction/loss of protein expression from that allele. If a gene’s remaining copy of the allele cannot compensate to preserve normal function, the gene is described as intolerant to haploinsufficiency, or the loss of an allele. We can predict tolerance to haploinsufficiency in many genes through the examination of the human genetic data, inferring tolerance based on the relative incidence of early truncation variants that occur in the general population versus the disease-associated population. If there are many ClinVar variants resulting in early truncations, with few early truncation variants in gnomAD, we can infer that the autosomal gene in question is intolerant to haploinsufficiency. In many cases, observing few or no early truncation variants in gnomAD data accompanied with early truncation ClinVar variants strongly indicates that the gene requires expression of both functional copies for normal biological function and is intolerant to haploinsufficiency.

A greater-than-expected percentage of NDD-associated genes are located on the X-chromosome [89,90]. Therefore, the loss of the allele on the X chromosome results in a strong male bias. In these cases, haploinsuffiency maybe be well tolerated in females resulting in mothers being carriers [13]. However, because males only possess one copy of the X chromosome, hemizygous males may be affected. Therefore, for sex-linked genes, interpretation of the databases can be confounded by the sex of the individual sequenced.

#### 3.2.2. Description of Exon Maps

Exon maps were created using the Matched Annotation from the NCBI and EMBL-EBI (MANE)-designated transcripts, with introns shortened (and therefore not to scale) for figure legibility. Early truncating variants were filtered from both ClinVar and gnomAD, searching for all mutations that result in early termination, specifically nonsense resulting in a stop codon and frameshifts, resulting in a downstream stop codon. The nucleotide position of these variants was then marked, either at the position of the mutated nucleotide (nonsense) or the first instance of insertion/deletion of a nucleotide (frameshift). Protein domain features are then depicted according to the nucleotides that code for them within the corresponding exon. The code and tutorial for creating these charts is available in our GitHub repository.

#### 3.2.3. NLGN1 and NLGN2 Are Tolerant to Haploinsufficiency

**Figure 1:**
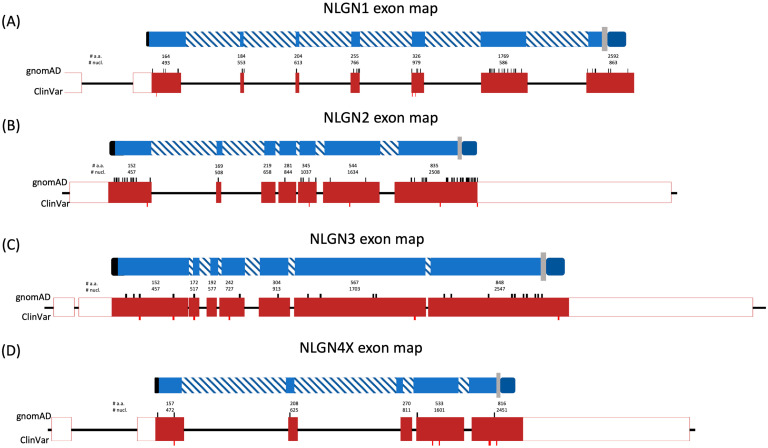
Early truncation variants are depicted on the exon maps of (**A**) NLGN1, (**B**) NLGN2, (**C**) NLGN3, and (**D**) NLGN4X, marked in black on top for gnomAD truncation variants, and in red on bottom for ClinVar early truncation variants. Atop the exon maps are the respective protein domain maps with gross structural features of NLGN1-4X, including signal peptide (black), extracellular domain (light blue), transmembrane domain (gray), and intracellular domain (dark blue). Intronic non-coded for regions of the protein alignment are depicted in blue stripes. The ending nucleotide and amino acid number are provided at the center of each exon. Due to varying intron size between the NLGN genes, introns were scaled down at a (**A**) 1:600, (**B**) 1:5, (**C**) 1:10, and (**D**) 1:70 ratio, respectively.

NLGN1 has 53 listed early truncation variants in gnomAD, all heterozygous, and only 3 early truncation variants in ClinVar, which are all before the last exon. These findings support a non-pathogenic phenotype for NLGN1 haploinsufficiency (Figure 1A). Additionally, NLGN2 has 64 listed gnomAD early truncation variants, all heterozygous, with two ClinVar disease-associated variants (Figure 1B). The numbers listed in the databases indicate it is likely that one can lose a single allele of NLGN1 or NLGN2 and not be impacted with NDDs.

#### 3.2.4. NLGN3 Is Tolerant to Haploinsufficiency in Females

For NLGN3, 9 of 20 gnomAD early truncation variants were found in the last exon, and only 1 was in a male patient. Conversely, 11 of 20 gnomAD early truncations were in exons other than the final exon, with only 1 male. Only three early truncations were found in ClinVar, two of which appeared before the last exon (Figure 1C). This female bias towards early truncation variation in gnomAD indicates an intolerance for males carrying early truncations of NLGN3. Additionally, the seeming tolerance for female heterozygosity points to the fact that NLGN3 is tolerant towards haploinsuffiency in females, but not complete loss of NLGN3, resulting in hemizygosity in males.

#### 3.2.5. NLGN4X Is Tolerant to Haploinsufficiency in Females

Out of 1195 listed NLGN4X gnomAD variants, 458 of those being missense variants, there are only five early truncation variants. Of these five truncation variants, four lie outside of the last exon in heterozygous females. Additionally, there are six NLGN4X truncation variants found in ClinVar, strongly implicating NLGN4X as intolerant to the loss of an allele in males (Figure 1D). For females, it is less clear. There are very few early truncations in gnomAD, but there is evidence from a pedigree that NLGN4X early truncations result in severe NDDs in the hemizygous males, with unaffected female carriers [13].

### 3.3. Analyses of Missense Variation

#### 3.3.1. Description of Algorithm

Analyzing a single gene at a time, we pull the list of genetic variants from both ClinVar (population with reported disease-associated variants) and gnomAD (variants in the population without reported disease). We filter for only missense variants that result in a change to a single amino acid in the coding region of the resultant protein. These variants are more likely to alter protein structure without leading to loss of the translation of the protein.

We perform a convolving algorithm on the two lists of variants that occur along the full span of the coding region.

**Figure 2:**
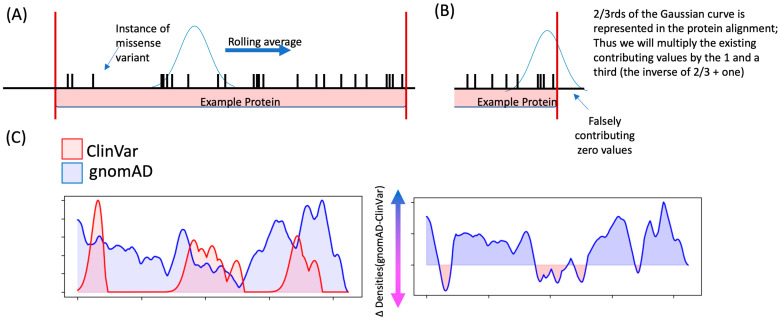
Diagrammatic explanation for determining clusters of sensitive residues along the span of a protein. (**A**) A rolling average is performed along the span of an example protein (in pink), with each instance of a missense variant having its location marked in black. For each residue within the protein, a tolerance value is calculated by summating nearby instances of missense variants multiplied by where they fall within the normal curve. (**B**) For residues near the far ends of the protein, the window of the normal curve will extend beyond the length of the protein, contributing false zeros. To correct for this, we multiply the calculated tolerance value by the percentage of the normal curve that falls outside the span of the protein. (**C**) We run this convolution algorithm on variants in both gnomAD and ClinVar and subtract the two curves to determine regions that are sparse in gnomAD variants while relatively more abundant in ClinVar variants.

1. Using a rolling average at each residue along the span of the protein, we calculate a “tolerance value” according to a normal distribution of nearby variants, not weighted for the number of individuals with a given variation (with a window size we have set to 31 residues) (Figure 2A). The rolling averages are discretely performed on the list of variants from ClinVar and gnomAD. This provides a curve with each point/residue along the protein representing an abstract tolerance value. Higher values in gnomAD represent residues that are highly tolerant (and present with a lot of variation within the human population), and higher values in ClinVar represent residues that are intolerant (thus many disease-associated variants appear within these spans of residues).
2. To correct for residues on the far ends of the protein, in which the Gaussian curve window would fall outside the range of the protein and thus contribute false zero values, we correct by multiplying by the inverse of the area under the curve to correct for the false zeros (Figure 2B). This falsely weights those variants within the distal ends, so we recommend exercising caution in interpreting tolerance values in these regions.
3. We normalize both curves such that they have the same maximum value (Figure 2C).
4. We invert the ClinVar curve (thus corresponding values are negative compared to gnomAD’s curve), then take the delta of the two curves by simply subtracting the values at each residue, collapsing the two curves into one (Figure 2C).

Negative values then correspond to regions enriched in ClinVar variants with relatively few gnomAD variants. Positive values represent regions that are more tolerant as defined by being sparse in ClinVar variation and abundant in gnomAD variation. We designate regions with negative values as sensitive, as those that are enriched with disease-associated variants over variants found in gnomAD.

In recent years, attention has been turned to developing methods for predicting the impact of missense mutations [91,92,93,94,95,96,97,98]. Although predicted loss-of-function mutations present high confidence predictive effects, 92% of missense variants within ClinVar were classified as variants with conflicting interpretations of pathogenicity [98]. These mixed classifications lead to confusion and are common because missense variance occurs at high frequencies within both unimpacted and disease-associated populations. Predictive algorithms have opted to utilize varying combinations of three overarching strategies: homologous regions/horizontal, linear/vertical, and structural/three-dimensional. Horizontal studies examine human variation within conserved domains of proteins to predict mutational constraints [99,100,101], whereas linear/vertical studies compare homologous or evolutionarily conserved genes to determine residue conservation [102]. Structural studies predict critical residues in the context of protein structure [95,96,97,98]. Our investigation into NLGN critical subdomains has opted for the horizontal approach in examining missense variation.

#### 3.3.2. Theory Behind Application

The utility of our algorithm is in bridging the gap between genetic analyses and the protein biology. Through filtering for and examining only missense variation, we can reveal sensitive regions of proteins at a subdomain level that likely do not disrupt total protein expression or function. This may allow for isolating the impact of a missense mutation on the broader signaling pathways. While this approach can benefit from comparison to known protein structures, one advantage to our approach is its non-reliance on structure. This allows researchers to identify previously unknown sensitive regions in portions of proteins with no known structure. For example, often the cytoplasmic tail of many membrane bound proteins, including NLGNs, are not included in protein structural studies.

The theoretical framework for these analyses can further inform therapeutic strategies for targeting the protein deficits, rather than treating broader symptoms of ASD. The goal is to elucidate regions of proteins from identified patients that cluster around critical protein domains and provide researchers with a guide to studying protein function. Discovering drugs that specifically rescue the function of the identified domain can provide patients therapeutics that address the underlying etiology and possibly address dysfunction in related proteins with variants in similar domains.

#### 3.3.3. Comparison of NLGNs

**Figure 3:**
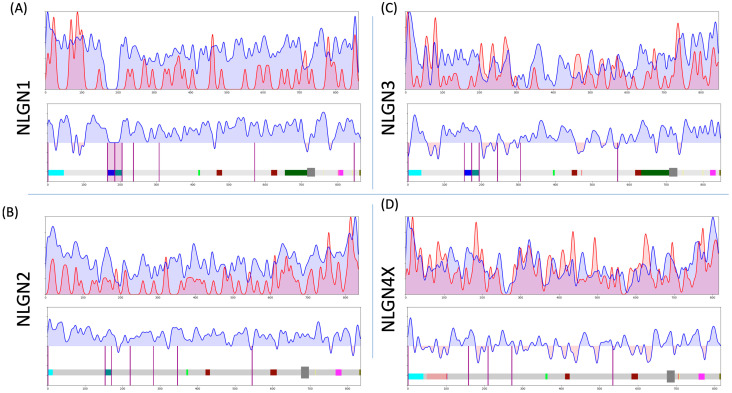
Tolerance curves of (**A**) NLGN1, (**B**) NLGN2, (**C**) NLGN3, and (**D**) NLGN4X. The top panel includes the 2 overlayed curves, ClinVar in red and gnomAD in blue, with the lower chart representing the delta of the 2 curves. The protein domain maps for the various NLGNs are provided at the bottom to assist correlating regional sensitivities to functional domains. (Sky blue is signal peptide, blue is A1 and A2 splice inserts, light green is NRXN binding interface, burgundy is the dimerization interfaces, dark gray is transmembrane domain, yellow is phosphorylation sites, pink is gephyrin-binding domain, and mustard is PDZ domain.) (**A**) The chart for NLGN1 does not include gnomAD variants from residue 165–205, the site of the 2 splice inserts. This is annotated with a pink shading over this missing region of unimpacted gnomAD variants. (**D**) The sensitive region from residue 50–100 of NLGN4X is shaded in red.

We applied the algorithm to four of the five NLGN genes (Figure 3), omitting NLGN4Y due to the scarcity of disease-associated variants (ClinVar only lists NLGN4Y p.R87Q, p.N401K). As a first pass at the validity of the algorithm, we evaluated NLGN4X (Figure 3D). Our group previously identified a cluster of ASD-associated missense variants around the 100 amino acid (a.a.) mark in NLGN4X [21]. We observed a trafficking deficit with the NDD-associated variants that we also observed in wild-type NLGN4Y. Furthermore, swapping p.P93 in NLGN4X with the analogous serine in NLGN4Y inhibited NLGN4X surface expression and rescued NLGN4Y surface expression, respectively. Therefore, we would expect that this region would be enriched in ClinVar variants using the algorithm. Strikingly, we did observe peaks in pathogenic variance above unimpacted variance in this region (Figure 3D). Similarly, we examined NLGN3 using the algorithm, specifically for detecting a sensitive region near NLGN3 p.R451, the most studied NLGN3 ASD-associated variant (p.R451C). Again, we found this variant was within a sensitive region (Figure 3C). It is possible that other focal regions of pathogenic variation might be obfuscated by baseline signal from the unimpacted gnomAD variant population, but for these examples, it was not the case.

Bolstered by our proof of concept in examining density of variants, we observed three regions that piqued our interest, due to their conservation between two or more *NLGN* genes. We have highlighted these regions, expounding on possible molecular and cellular deficits caused by reported pathogenic variants within these regions.

### 3.4. Sensitive Regions

#### 3.4.1. Region 1

**Figure 4:**
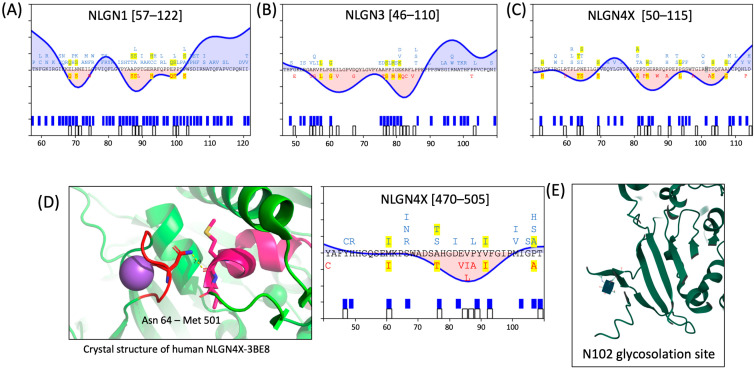
An N-terminal critical region of (**A**) NLGN1, (**B**) NLGN3, and (**C**) NLGN4X, spanning from ~50–110 a.a. Analogous residue alignments are depicted between NLGN1, NLGN3, and NLGN4X, with gnomAD and ClinVar missense variants in blue and red, respectively. Missense variants that are common to both are highlighted in yellow. (**D**) The crystal structure of NLGN4X reveals the 64th asparagine, found within the first dip of this critical region, interacts with methionine 501 which is near another sensitive region of NLGN4X. (**E**) The second dip within this critical region is near an identified N-linked glycosylation site at N102.

We identified a region enriched in ASD-associated variants in NLGN4X around the 100th a.a. Importantly, analysis of missense variation in both NLGN1 and NLGN3 also implicates this region as crucial for proper protein function (Figure 4A–C). NLGN1 has eight missense variants in this region found in ClinVar (p.A87S, p.P88S, p.P89L, p.T90R, p.R93H, p.E99Q, p.P100S, p.P103S). NLGN3 has 7 missense variants in this region found in ClinVar (p.A76T, p.P77S, p.I77M, p.E81K, p.K82Q, p.R83C, p.L85V), and NLGN4X has 11 missense variants in this region (p.P81A, p.T83P, p.G84R, p.R87W, p.P90A, p.P94L, p.T98A, p.T103A, p.T104S, p.A108G, p.L114P). Indeed, one study examined a critical residue within this region in NLGN1, in which the ASD-associated NLGN1 p.P89L variant caused a 50% and 30% reduction in NLGN1 expression in a homozygous and heterozygous mouse model, respectively [34]. Another set of recently published studies examined the NLGN1 p.T90R variant [36,37], which has not yet been deposited into ClinVar.

Within this cluster, we observed two sensitive peaks conserved across the three NLGNs of interest, consistent with two discrete critical subdomains. Included within the first peak in NLGN1 (70-NNEIL-74) are the variants p.E68Q, p.N70S, and p.L74W. For NLGN3, the first peak (59-PSEIL-63) includes p.A54T, p.R55G, p.P57L, p.S60G, and p.I62V. Similarly, NLGN4X has a homologous stretch (63-PNEIL-67) that includes p.R59S, p.P63T, p.N64S, and p.P69S. Each isoform has a conserved XXEIL motif, which includes disease-associated variants (Figure 4A–C). Examining the protein structure of residues in this XXEIL motif reveals that they interact with residues within a sensitive region found in NLGN3 (525–540) and NLGN4X (490–498), as revealed by the crystal structure of NLGN4X (Figure 4D) [103]. The second sub-domain within this region contains variants characterized in Nguyen et al. 2020, that display a trafficking deficit [21]. These variants might disrupt the N-linked glycosylation site, which may be crucial for proper folding and surface trafficking of NLGN4X [32] (Figure 4E). This region is also sensitive to variation in NLGN1 and NLGN3, although N-linked glycosylation has not been studied at the analogous NLGN1 p.N109 and NLGN3 p.N98 sites.

#### 3.4.2. Region 2

**Figure 5:**
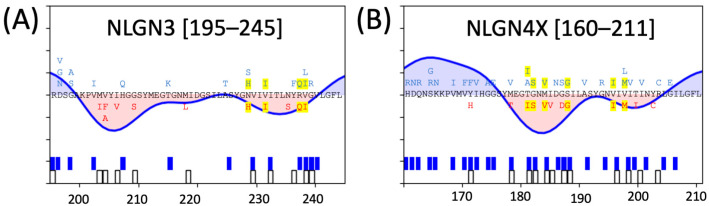
The second observed critical cluster in (**A**) NLGN3 and (**B**) NLGN4X. Analogous residue alignments are depicted between NLGN3 and NLGN4X, with gnomAD and ClinVar missense variants in blue and red, respectively. Missense variants that are common to both are highlighted in yellow.

NLGN3 and NLGN4X have analogous sensitive regions ~200th a.a. (Figure 5). This region is of particular interest because it reveals a pronounced dip in gnomAD density in all NLGN isoforms, with NLGN3 and NLGN4X having indicated intolerance values along this span of residues (195–245 and 160–211, respectively). Within this region, NLGN3 has 11 ClinVar missense variants and NLGN4X has 12 ClinVar missense variants.

#### 3.4.3. Region 3

**Figure 6:**
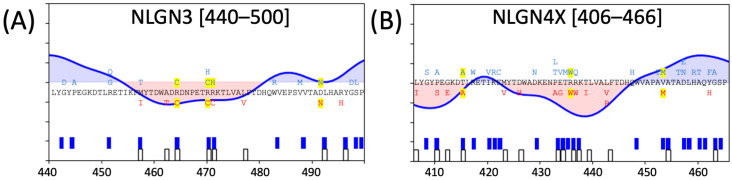
The third observed critical cluster in (**A**) NLGN3 and (**B**) NLGN4X, around the ~450 a.a. region of NLGN3. This is the region in which the NLGN3 R471C (R451C in most of the literature due to alternative splicing) is found. Analogous residue alignments are depicted between NLGN3 and NLGN4X, with gnomAD and ClinVar missense variants in blue and red, respectively. Missense variants that are common to both are highlighted in yellow.

One source of validation of the principle behind these analyses was the detection of a sensitive region around a.a. 450–500 of NLGN3 and NLGN4X (Figure 6). The NLGN3 p.R451C variant has been shown to be ER-retained, causes gain-of-function effects in excitatory synaptic transmission in certain models, and leads to a 90% reduction in NLGN3 expression in hemizygous male mouse models [22,26,27]. Despite the proximity of this variant to the NLGN dimerization interfaces, in vitro studies demonstrated NLGN3 p.R451C can still dimerize with NLGN1 and WT NLGN3, suggesting that dimerization is not the cause of NLGN3 p.R451C dysfunction [46]. In vivo studies in hemizygous mice find gain-of-function effects in both learning and memory behavioral testing, as well as circuit-specific increases in inhibitory synaptic events [27,47,52]. Indeed, overexpression of NLGN3 p.R451C in heterologous cells causes ER-retention of the protein, along with upregulation of the ER stress response, measured through increased expression of BiP and CHOP [53]. Additionally, secondary structural characterization of NLGN3 p.R451C via mass spectrometry indicates the mutated cysteine does not form a disulfide bond with free cysteines in NLGN3, but rather might act as an ER-retention signal as posited in Comoletti et al. 2004 [26]. The adjacent NLGN3 p.R470 also has a cysteine ClinVar submission, and a nearby p.R464C variant, potentially implicating these variants as susceptible to similar deficits. NLGN4X also displays sensitivity to mutations in its analogous region, with a reported p.R437W missense variant, which is analogous to NLGN3 p.R471 (most often referred to as p.R451 in the literature, which differs by an alternatively spliced insert).

Additionally, NLGN4X displays another intolerant region around 70 residues upstream (Figure 3D) with an analogous intolerant region conserved in NLGN1. Interestingly, this region coincides with the NRXN binding interface of NLGN1 and NLGN4X, suggesting that these variants might disrupt NRXN affinity (Figure 3A,D). This region does appear to be sparser in gnomAD variation in NLGN2 and NLGN3 (Figure 3B,C), although they do not have reported ClinVar variants that would lead to an intolerance value using this algorithm.

#### 3.4.4. Additional Sensitive Regions

##### Juxtamembrane Cleavage Domain

Cleavage of NLGN1 and NLGN3 has been previously studied [104,105,106] with important functional implications of the extracellular domain (ECD) cleavage byproduct of NLGN3 serving as a mitogen for glioblastoma in the brain [107]. Therefore, NLGN ECDs potentially have broader effects in the brain, and this cleavage event could prove more functionally important than simply proteostasis.

The juxtamembrane domain of all NLGNs is defined by its purportedly heavily O-glycosylated span, which in certain NLGN genes is recognized and cleaved by proteases. Within NLGN1, there are four ClinVar missense variants within this span (p.T667K, p.Q675R, p.P678A, and p.T694A), in a region that is relatively sparse of gnomAD variants, from 673–699 a.a. (p.T674I, p.V678I, p.A681S, p.A681V, p.V682A, p.L686M, p.N690H, p.I691M, p.L692M, p.A695T, p.Y698F, p.Y698C). This means there are only 12 variants in a 26-residue span, while the gnomAD sensitivity curve drops ~1/3rd from its peak. This region is highly divergent between NLGNs, but there are corresponding sensitive regions, suggesting a per gene functional importance to the region or functional dependence on structure or posttranslational modifications beyond pure sequence identity. In NLGN2, there are five reported ClinVar missense variants (p.R641P, p.R642W, p.P645L, p.E654K, p.P655T), with several of those variants found within a proline-rich region of NLGN2 spanning from residue 643 to 659 (PPPPATLPPEPEPEP). In NLGN3, there are five variants within this same region (p.T652A, p.T659N, p.R671W, p.I674T, p.N680D), and NLGN4X has six ClinVar variants found within this region (p.S620L, p.T625I, p.T625N, p.R626Q, p.R627Q, p.W633R).

Interestingly, this span of sensitive residues occurs near two O-glycosylation sites in NLGN1, p.S703 and p.S706, as discovered through mass spectroscopy analysis [108]. Previous work characterizing NLGN1 cleavage posited that these O-glycan groups act as a protective group preventing cleavage, as 4× alanine mutations at these residues dramatically increase NLGN1 cleavage [106]. Perhaps divergent O-glycosylation [105] in this region determine NLGN3’s isoform-specific activity-dependent cleavage phenotype. More broadly, do variants that disrupt O-glycosylation and/or cleavage result in NDDs?

##### Differential Sensitivity in the Proline-Rich NLGN2 C-Tail

NLGN2 contains a proline-rich cytoplasmic tail, allowing for its preferential binding to inhibitory synaptic scaffolding proteins and localizing it to inhibitory synapses [109,110]. We observe unique pathogenic variants present in the c-tail of NLGN2, that are tolerant in other NLGNs. This difference might reveal NLGN2-specific mechanistic insight. Perhaps these variants disrupt NLGN2’s trafficking to inhibitory synapses. It would be interesting to see if patients with mutations in these regions present with distinct clinical phenotypes indicative of a disruption to inhibitory synapse physiology.

## 4. Conclusions

Within this review, we summarize the literature for rare variants in NLGNs and provide a framework for determining sensitive regions of proteins by applying a rolling average to highlight clustered regions of pathogenic variants. Based on empirical observations that NLGN ASD-associated variants clustered in regions relatively devoid of non-pathogenic variation, we developed an approach to systematically evaluate any gene. Using the newly developed algorithm, we specifically investigate NLGNs, finding several conserved critical regions across the *NLGN* gene family.

*NLGN3* and *NLGN4X* show the highest degrees of sensitivity, likely because they are sex-linked and most mutations in autosomal genes are well tolerated in heterozygous individuals. This tolerance of the loss of an allele in autosomal NLGNs is further evinced when examining early truncation data, with very few early truncation variants appearing in gnomAD for NLGN3 and 4X in males, indicating their intolerance to haploinsufficiency. In contrast, gnomAD is rife with early truncations in both autosomal *NLGN1* and *NLGN2* genes. We further discuss three regions that analyses reveal as critical for NLGN function. Within the 50–100 a.a. region, we observe two distinct subdomains conserved across NLGN1, 3, and 4X. Examining the crystal structure of NLGN4X indicates the first critical subdomain directly interacts with the top of the α-helix span ~560 a.a., another region enriched in disease-associated variants in NLGN3 and NLGN4X. The second sub-region includes variants in NLGN4X we previously characterized as having trafficking deficits, with an important N-linked glycosylation site (N102) nearby [21,32]. We also discuss another sensitive region at ~200 a.a. region found in NLGN1, 3, and 4X. This region is revealed to be enriched in ClinVar variants compared to gnomAD variants in NLGN3 and 4X using the comparative analysis. The analogous region is relatively sparse in gnomAD variants in NLGN1 and NLGN2. The conservation of this region across NLGNs indicates this region is structurally important for proper NLGN function. However, it is relatively unexplored, leaving room for further research. Lastly, we discuss the well-studied NLGN3 p.R451C (MANE annotation: p.R471C) variant and postulate on how accompanying nearby ClinVar variants in NLGN1, NLGN3, and NLGN4X might also cause similar cellular distress as the protein trafficking is disrupted in the ER [53].

Our approach can be broadly utilized for analysis in other genes and provides several advantages over legacy approaches or newer multimodal approaches. Through simply utilizing human genetic data, we avoid confounding the data with evolutionarily similar genomes that might lose features/sequences that are critical and unique to human biology. Additionally, this approach does not utilize protein structure to model tolerance and loss of protein function, thus we can reveal critical regions in unstructured and uncharacterized parts of proteins, like the cytoplasmic tail of NLGNs. This approach gave us insight into the importance of the uniquely proline-rich cytoplasmic tail of NLGN2, highlighting it for further biological exploration. We hope our approach provides molecular biologists studying disease-associated variants a framework to guide their studies on the biological impacts using human genetic data as a guide.

## Figures and Tables

**Table 2:** Summary of reported NLGN2 variants.

Gene	Variant	Patient Clinical Presentation	Predicted Loss of Allele	Functional Effects in Culture	Mouse Model Phenotypes	Citation
NLGN2	Y147*	ASD/ID, anxiety, hyperphagia, obesity	Yes	-	-	[40]
	R215H	SCZ	No	Decreased neurexin aggregation; increased ER-retention; impaired synaptogenesis and decreased sIPSCs	Decreased protein expression; impaired synaptogenesis; decrease in mIPSC amplitude and frequency and altered E/I ratio; increased anxiety behavior	[23,24,25]
	V510M	SCZ	No	Unaffected neurexin aggregation surface expression ER-retention, synaptogenesis, and sIPSCs	-	[23]
	R621H	SCZ	No	Unaffected neurexin aggregation, surface expression, ER-retention, synaptogenesis, and sIPSCs	-	[23]
	A637T	SCZ	No	Unaffected neurexin aggregation, surface expression, ER-retention, synaptogenesis, and sIPSCs	-	[23]

## Data Availability

Code used for identifying and visualizing sensitive regions of genes/proteins can be found at our GitHub repository (https://github.com/AlexanderWLehr/Lehr-etal-Gene-2024, accessed on 5 November 2024).
